# Death of Dr. R. A. T. Ridley

**Published:** 1872-02

**Authors:** 


### MISCELLANEOUS.

Death of Dr. Robert A. T. Ridley.—It is with a sad heart we chronicle the death of Dr. R. A. T. Ridley, which occurred at his residence, at eight o'clock on Wednesday morning last. His death was sudden and unexpected. On Monday, he was on the streets, in the regular pursuit of his profession; but on Monday night he was attacked with a hemorrhage of the stomach and bowels, which was the cause of his death.

Dr. Ridley was born in Granville county, North Carolina, March 5th, 1806, and graduated at Chapel Hill, in that State. He came to Georgia in 1828, and settled at Hillsboro, Jasper county, where he read medicine with his brother, Dr. Charles Ridley, and took his medical degree at Charleston, South Carolina. While residing at Hillsboro, he was married to Miss Mary E. Morris, who still survives him.

In 1838, Dr. Ridley came to LaGrange, where he has practiced his profession ever since, with the exception of an intermission of a year or two, when he retired from the practice. The results of the war made it necessary for him to resume his profession, and it may be literally said that he died in its practice. He was one of the finest physicians in the State, and was recognized as a leading practitioner wherever he was known. Perhaps no physician had more of the confidence of the afflicted than he.

Dr. Ridley has several times represented this county in both branches of our State Legislature, where he always proved himself to be a safe legislator, and the worthy peer and associate, intellectually, of the best men in the State. Impulsive and warm-hearted, honest and sincere in all he did, with an intellect clear and quick, forcible in discussion, and ready at repartee, he was a man of mark in our Legislature when such men as Andrew J. Miller, Charles J. Jenkins, and others of their calibre, made up the leading spirits of our law-makers. Incorruptible, he leaves a record of honesty and fidelity to truth and justice that all may imitate. True to his country, he was a patriot whose devotion was pure and sacred. A humane man, he sympathized with all who were in trouble. True to his country and his kind, he was true to his Maker, and died in the full assurance of his acceptance with Him who gave and He that taketh away. May the sod rest lightly upon his new-made grave!

Dr. Ridley leaves a fond and devoted wife and four affectionate sons to mourn their irreparable loss. To them and his aged mother in Israel, now bordering on her four-score years and ten, residing with the family, and to the relatives generally, we offer for ourselves, and in behalf of the whole community, heart-felt condolence, and to the memory of our departed friend the tribute of love and respect due to a true man and a kind neighbor, whose soul has gone to that "bourne from whence no traveler e'er returns."—*LaGrange Reporter, December* 21, 1871.

